# Emerging therapies for small cell lung cancer

**DOI:** 10.1186/s13045-019-0736-3

**Published:** 2019-05-02

**Authors:** Sen Yang, Zhe Zhang, Qiming Wang

**Affiliations:** Department of Internal Medicine, The Affiliated Cancer Hospital of Zhengzhou University, Henan Cancer Hospital, Zhengzhou, 450008 China

**Keywords:** Small cell lung cancer, Chemotherapy, Immunotherapy, Targeted therapy

## Abstract

Currently, chemotherapy remains the standard treatment for first- and second-line management of small cell lung cancer (SCLC). Immunotherapy has made progress in the treatment of SCLC, and nivolumab, pembrolizumab, atezolizumab, and durvalumab have led to significant improvements in clinical outcomes of SCLC. Regarding options in other classes of therapy, the cytotoxic drug lurbinectedin was granted orphan drug status based on a remarkable objective response rate of 39.3%. In addition, an increase in progression-free survival (PFS) was achieved in a phase II study of anlotinib (ALTER 1202). Future prospects for even better outcomes in SCLC lie in novel ways to integrate immunotherapy and small-molecule TKI drugs. Innovative clinical trial designs are needed to efficiently explore the increasing number of options with new drugs and new combinations thereof for SCLC.

## Introduction

Small cell lung cancer (SCLC) accounts for approximately 15% of all lung cancer and is the leading cause of cancer death among men and the second leading cause of cancer death among women worldwide [1, 2]. The prognosis of patients with SCLC is dismal with a 5-year survival rate of less than 5% and an average overall survival period of only 2–4 months for patients not receiving any active treatment [3, 4]. The primary risk factor for SCLC remains smoking tobacco, which is also associated with high mutation burden in this disease [5]. Early detection of SCLC is challenging due to the lack of specific symptoms and rapid tumor growth, making current approaches to screening ineffective in diagnosing patients at early disease stages.

According to the veteran affairs lung group staging criteria, SCLC is divided into limited and extensive stages. The extensive stage accounts for approximately 65% of new cases [6]. Therapeutic options for SCLC are limited. Surgery in the form of lobectomy is a potential option for TNM stage I (T1-2N0M0) without mediastinal or supraclavicular involvement [7]. First-line standard chemotherapy is a combination of etoposide or irinotecan with platinum. In the limited stage, concurrent or sequential radiotherapy to the thorax and mediastinum is also needed. If a complete response was achieved, prophylactic cerebral irradiation (PCI) is indicated to prevent the subsequent development of metastasis to the brain. In the extensive stage, chemotherapy is the mainstay treatment in the first-line setting. The value of thoracic radiation and PCI is controversial, however, and is not a standard recommendation for all patients [8, 9]. The median overall survival (OS) for extensive stage SCLC patients treated with standard frontline chemotherapy is only approximately 10 months [10, 11]. SCLC is usually sensitive to the initial treatment; however, most patients develop recurrent disease, often with additional sites of metastasis after initial treatment [12]. Unfortunately, very few drugs are approved as effective for second-line treatment of SCLC. Topotecan is a standard second-line choice but is not uniformly used for patients in part due to its modest efficacy and significant hematologic toxicity. Overall survival (OS) in patients treated with topotecan is only 26 weeks vs. 14 weeks in patients managed with the best supportive care alone [13]. Due to the modest efficacy of available conventional salvage treatments as measured by rates of PFS and OS, the quest for more effective therapeutic approaches has not abated [14]. Single-agent regimens of standard cytotoxic agents, including paclitaxel, docetaxel, gemcitabine, and vinorelbine, have been studied in phase II clinical trials as second-line therapies with modest results. In more recent years, targeted therapy and immunotherapy have also been actively tested with many disappointments but also some encouraging results. Here, we review the results of recent clinical evaluations of new treatment strategies for SCLC with emphasis on agents having the most promise to change the prognosis of this disease.

## Chemotherapy

### Metronomic chemotherapy

Metronomic chemotherapy has gained increased attention in recent years. A metronomic chemotherapy regimen of cisplatin, etoposide, and irinotecan was compared to single-agent topotecan in sensitive recurrent SCLC (JCOG0605) in Japanese patients. The study enrolled 180 patients and randomized patients 1:1 to the control or metronomic regimen. OS in patients taking the three-drug metronomic regimen was significantly longer than for patients treated with topotecan alone (18.2 vs. 12.5 months, HR 0.67, *P* = 0.0079) [15]. This very positive result represents an important breakthrough in the second-line therapy for SCLC. However, the toxicity of the three-drug metronomic regimen cannot be ignored. Whether metronomic chemotherapy could be a second-line treatment option in the future remains to be explored and studied in additional patient populations.

### Lurbinectedin

Lurbinectedin is an inhibitor of RNA polymerase II, which is commonly hyperactivated in SCLA, resulting in excessive transcription in tumor cells. Inhibition by lurbinectedin is expected to decrease tumor cell proliferation primarily by inhibiting mitosis [16]. The United States Food and Drug Administration (FDA) granted lurbinectedin (PM1183) orphan drug status for the treatment of SCLC. This designation was based on a phase II multicenter basket study (NCT02454972) that assessed efficacy in 68 recurrent SCLC patients. Among the 61 patients evaluable for efficacy, ORR was 39.3%, 7 patients had stable disease for more than 4 months after treatment, the overall clinical benefit rate was 50.8%, the rate of disease control was 73.8%, and median OS was 11.8 months. The most common adverse event was myelosuppression: 44% neutropenia grade (G) 3/4, 12% febrile neutropenia, and 8% thrombocytopenia G 3/4. Among these adverse events, eight patients experienced dose delay due to neutropenia G2-4, and ten patients had their dose reduced due to neutropenia G4 (Table 4) [17]. An ongoing phase III trial of lurbinectedin plus doxorubicin vs. topotecan has completed accrual and should provide additional evidence in support of the efficacy of this agent in SCLC.

## Immunotherapy

### Ipilimumab

Cytotoxic T lymphocyte antigen-4 (CTLA-4) is a negative regulator of the priming phase of T cell activation and a validated target for anticancer therapy [18–21]. Ipilimumab is a human anti-CTLA-4 monoclonal antibody that blocks CTLA-4 and its ligands (CD80/CD86), promoting activation and proliferation of T cells [22]. Ipilimumab in early clinical trials has shown durable inhibition in multiple tumor types [23–25]. Based on data from previous clinical studies, an initial phase II study evaluated the safety and efficacy of ipilimumab in combination with carboplatin and etoposide as first-line chemotherapy for patients with extensive stage SCLC (Table 1). In this trial, 42 patients were enrolled, and 72.4% of patients achieved an objective response, while 84.8% achieved an immune-related objective response. Median progression-free survival (PFS) was 6.9 months (95% CI 5.5–7.9), and median immune-related PFS was 7.3 months (95% CI 5.5–8.8). Median OS was 17.0 months (95% CI 7.9–24.3). At least one G 3 or higher toxicity developed in 35 of 39 patients (89.7%); in 27 patients (69.2%), this was related to ipilimumab. Additionally, five deaths were reported as related to ipilimumab. G 3 or higher toxicities were primarily neurological adverse reactions (AEs) (10.3%), diarrhea (48.7%), neutrophil count decrease (23.1%), anemia (15.4%), infection (28.2%), and sepsis (10.3%) (Table 4) [26]. Another phase II study was conducted to test ipilimumab in combination with paclitaxel and carboplatin. This study enrolled 130 patients, and 128 patients were treated. Patients were randomized 1:1:1 to receive paclitaxel + carboplatin + placebo (control), ipilimumab + paclitaxel + carboplatin followed by placebo + paclitaxel + carboplatin (concurrent ipilimumab), or placebo + paclitaxel + carboplatin followed by ipilimumab + paclitaxel + carboplatin (phased ipilimumab). The best overall response rate (BORR) in control, concurrent, and phased ipilimumab treatments was 49%, 32%, and 57%, respectively, while immune-related BORR was 53%, 49%, and 71%, respectively. PFS of control, concurrent, and phased ipilimumab was 5.2, 3.9, and 5.2 months, respectively, and immune-related PFS was 5.3, 5.7, and 6.4 months (HR = 0.75, 0.64; *P* = 0.11, 0.03), respectively. Median OS for these three cohorts was 9.9, 9.1, and 12.9 months (HR = 0.95, 0.75; *P* = 0.41, 0.13), respectively. The incidence of treatment-related G 3/4 AEs appeared more commonly in ipilimumab-containing arms (concurrent, 43%; phased, 50%) than in the control arm (30%). G 3 or higher toxicities were primarily ALT (18%) and AST (13%) vs. fatigue (12%), arthralgia (10%), diarrhea (10%), neutropenia (10%), and anemia (10%) (Table 4) [27]. The results of these phase II studies indicated that ipilimumab combination with chemotherapy might improve outcomes for patients with untreated extensive stage SCLC. A confirmatory phase III clinical trial of ipilimumab, etoposide, and platinum vs. placebo, etoposide, and platinum was performed. A total of 1132 patients were enrolled, and 954 were treated. BORR was identical in the two cohorts at 62%. Median PFS was 4.6 months in the ipilimumab arm compared to 4.4 months in the placebo group (HR, 0.85; *P* = 0.016). However, there was no significant difference in median OS between the two groups at 11.0 and 10.9 months (HR, 0.94; *P* = 0.38) for ipilimumab and placebo arms, respectively. Rates and severity of treatment-related adverse events were similar between the arms, except for diarrhea, rash, and colitis, which were more frequent in chemotherapy plus ipilimumab. Five treatment-related deaths occurred with chemotherapy plus ipilimumab and two with chemotherapy plus placebo. G 3 or higher toxicities were primarily neutropenia (24%) and anemia (11%) vs. neutropenia (14%) (Table 4) [28].

**Table 1:** Completed immunotherapy clinical trials in ES-SCLC

Phase	Study	Treatment arms	Patients (*n*)	ORR (%)	PFS (months)	OS (months)
First line						
II	NCT01331525	Ipilimumab + carboplatin + cisplatin; maintained with ipilimumab	42	72.4	6.9	17.0
II	CA184-041	Ipilimumab/placebo + carboplatin + paclitaxel vs. placebo/ipilimumab + carboplatin + paclitaxel vs. placebo + carboplatin + paclitaxel; maintained with ipilimumab vs. placebo	128	32 vs. 57 vs. 49	5.7 vs. 6.4 vs. 5.3^*^ (HR = 0.75, 0.64) (*P* = 0.11, 0.03)	9.1 vs. 12.9 vs. 9.9 (HR = 0.95, 0.75) (*P* = 0.41, 0.13)
III	CA184-156	Ipilimumab + etoposide + cisplatin/carboplatin vs. placebo + etoposide + cisplatin/carboplatin; maintained with ipilimumab vs. placebo	954	62 vs. 62	4.6 vs. 4.4 (HR = 0.85) (*P* = 0.016)	11 vs. 10.9 (HR = 0.94) (*P* = 0.38)
III	Impower-133	Atezolizumab + carboplatin + etoposide vs. placebo + carboplatin + etoposide; maintained with atezolizumab vs. placebo	403	60.2 vs. 64.4	5.2 vs. 4.3 (HR = 0.77) (*P* = 0.02)	12.3 vs. 10.3 (HR = 0.70) (*P* = 0.007)
Maintenance						
II	NCT02359019	Pembrolizumab	45	11.1	1.4	9.6
Relapsed						
I/II	CheckMate-032	Nivolumab 3 mg/kg vs. nivolumab 1 mg/kg + ipilimumab 3 mg/kg vs. nivolumab 3 mg/kg + ipilimumab 1 mg/kg	213	10 vs. 23 vs. 19	1.4 vs. 2.6 vs. 1.4	4.4 vs. 7.7 vs. 6.0
IB	KEYNOTE-028	Pembrolizumab	24	33.3	1.9	9.7
II	KEYNOTE-158	Pembrolizumab	107	18.7	2.0	9.1
I/II	NCT02261220	Durvalumab + tremelimumab	30	13.3	1.8	7.9

^*^irPFS

### Atezolizumab

Atezolizumab is a humanized monoclonal antibody that targets programmed death ligand 1 (PD-L1), an inhibitory ligand that negatively regulates T cell activation and proliferation by binding to the PD-1 receptor [29]. An initial phase I study established that atezolizumab monotherapy had acceptable side effects with promising durability of response in patients with relapsed SCLC [30]. The Impower133 study (Table 1) is a phase III trial that evaluated the combination of atezolizumab with etoposide and carboplatin vs. placebo combined with platinum doublet in untreated extensive stage SCLC patients. Patients without disease progression at the end of four cycles of combination treatment continued to receive maintenance atezolizumab or placebo. A total of 403 patients were enrolled and randomly assigned to either the atezolizumab group or the placebo group in a 1:1 ratio. The objective response rate was 60.2% with the addition of atezolizumab and 64.4% for the placebo plus platinum doublet group. Median PFS was 5.2 and 4.3 months, respectively, (hazard ratio [HR], 0.77; 95% confidence interval [CI], 0.62 to 0.96; *P* = 0.02) in favor of atezolizumab. Median OS was also superior with atezolizumab at 12.3 months vs. 10.3 months (HR, 0.70; 95% CI, 0.54 to 0.91; *P* = 0.007). This study established a significant improvement in efficacy for extensive stage patients treated with atezolizumab plus standard carboplatin and etoposide regimen in frontline treatment. The most common G 3 or 4 adverse events related to the trial regimen were neutropenia, anemia, and decreased neutrophil count. Deaths related to the trial regimen occurred in three patients (1.5%) in the atezolizumab group (death was due to neutropenia in one patient, pneumonia in one patient, and an unspecified cause in one patient) and in three patients (1.5%) in the placebo group (death was due to pneumonia in one patient, septic shock in one patient, and cardiopulmonary failure in one patient). G 3 or higher toxicities were primarily neutropenia (22.7%), anemia (14.1%), decreased neutrophil count (14.1%), and thrombocytopenia (10.1%) vs. neutropenia (24.5%), anemia (12.2%), and decreased neutrophil count (16.8%) (Table 4) [31].

### Pembrolizumab

Pembrolizumab is a humanized monoclonal antibody that binds the PD-1 receptor, inhibiting the negative signaling induced by the interaction between PD-1 and its ligands [32]. KEYNOTE-028 was a phase Ib trial conducted to evaluate the safety and efficacy of pembrolizumab in 24 recurrent SCLC patients with PD-L1 positive tumors. The most common adverse events were asthenia, fatigue, and cough. Only two patients experienced G 3–5 treatment-related AEs: one bilirubin elevation and one colitis. Objective response was recorded in eight patients for an ORR of 33% (Table 1). KEYNOTE-028 indicated that the safety of pembrolizumab in SCLC was consistent with data in other tumor types, and pembrolizumab demonstrated promising antitumor activity in patients with pretreated SCLC. Treatment-related AEs were observed in 16 of 24 patients (66.7%). Two patients experienced G 3 to 5 treatment-related AEs: one patient had G 3 bilirubin elevation and one patient had G 3 asthenia and G 5 colitis. No G 3 to 5 treatment-related AEs occurred in over 10% of participants (Table 4) [33]. A larger phase II study tested pembrolizumab in recurrent SCLC patients regardless of PD-L1 status. ORR was 18.7% for the entire group and 35.7% in patients with PD-L1 positive tumors. Median PFS and OS were 2.0 months and 9.1 months, respectively, for the entire group. In PD-L1 positive patients, PFS was 2.1 months, but OS improved to 14.6 months. In contrast, PFS and OS were 1.9 and 7.7 months, respectively, in PD-L1 negative patients. Treatment-related AEs occurred in 63 patients (59%), resulting in 4 discontinuations and 1 death (pneumonia). ORR was 18.7% (20/107) overall, 35.7% (15/42) in patients with PD-L1-positive tumors, and 6.0% (3/50) in patients with PD-L1-negative tumors. Median PFS was 2.0 months for all patients, 2.1 months in patients with PD-L1-positive tumors, and 1.9 months in patients with PD-L1-negative tumors. Median OS was 9.1 months overall, 14.6 months in patients with PD-L1-positive tumors, and 7.7 months in patients with PD-L1-negative tumors (Table 4) [34]. The most recent data of KEYNOTE-028 and KEYNOTE-158 came from AACR Annual Meeting 2019. In the pooled analysis, 83 were eligible for efficacy analyses—the objective response rate (ORR) was 19.3%, which included 2 complete responses and 14 partial responses. The median duration of response (DOR) was not reached at the time of this analysis. Of the 16 responders, 9 had responses lasting for at least 18 months. After a median of 7.7 months of follow-up, the median progression-free survival (PFS) was 2 months and median overall survival (OS) was 7.7 months. At 12 months, PFS and OS were 17% and 34%, respectively, and at 24 months, PFS and OS were 13% and 21%, respectively [35]. Pembrolizumab was also tested as a maintenance therapy for extensive stage SCLC patients who did not progress upon the completion of frontline chemotherapy. This study enrolled 45 patients, 5 of whom achieved an objective response, resulting in an ORR of 11.1%. Median PFS was remarkably short at only 1.4 months, and OS was 9.6 months. Overall, pembrolizumab exhibited promising efficacy for recurrent SCLC, particularly in patients with PD-L1 positive tumors. The most common adverse events were fatigue, nausea, cough, and dyspnea. One patient experienced atrioventricular conduction block, and one patient developed type 1 diabetes (Table 4) [36].

### Nivolumab

Nivolumab is a fully human PD-1 immune checkpoint inhibitor antibody with proven safety and efficacy in patients with SCLC [37, 38]. Preclinical data also suggested an improved anticancer activity for combined PD-1- and CTLA-4-targeted antibodies, and the combination of nivolumab and ipilimumab demonstrated durable responses in several tumor types [39–42]. CheckMate-032 was initially designed as a basket phase I/II study to evaluate the safety and activity of nivolumab as a monotherapy or in combination with ipilimumab in several tumor types. A total of 216 patients were enrolled, and 213 were treated. SCLC patients who had previously failed platinum-based chemotherapy were treated with the single-agent nivolumab or a combination of different doses of nivolumab and ipilimumab. ORR was 10% for the single-agent nivolumab dosed at 3 mg/kg, whereas ORR was 23% for the combination of nivolumab dosed at 1 mg/kg along with 3 mg/kg ipilimumab. Moreover, ORR was no better at 19% in the cohort treated with 3 mg/kg nivolumab combined with 1 mg/kg ipilimumab. Median PFS was 1.4, 2.6, and 1.4 months for nivolumab alone, 1 mg/kg nivolumab + 3 mg/kg group ipilimumab, and 3 mg/kg nivolumab + 1 mg/kg group ipilimumab, respectively. Similarly, OS was 4.4, 7.7, and 6.0 months, respectively. In terms of safety, the most common G 3–4 adverse events were increased lipase and diarrhea. No G 3 to 5 treatment-related AEs occurred in more than 10% of participants (Table 4) [43]. The results of the expanded cohort of recurrent SCLC patients treated with nivolumab (1 mg/kg) with or without ipilimumab (3 mg/kg) showed significant efficacy for this unmet need in recurrent patients, leading to the inclusion of this regimen in guideline treatment recommendations for US patients and regulatory approval by the US FDA for single-agent nivolumab as a salvage regimen for SCLC.

### Durvalumab

Durvalumab is another humanized monoclonal antibody that targets programmed death ligand 1 (PD-L1). There are only a few studies on durvalumab in SCLC. A phase I study to evaluate the safety and clinical activity of durvalumab in combination with tremelimumab in extensive disease small-cell lung cancer was performed in 2017. In this study, 30 patients in the expansion phase received treatment, and 20 patients reported over 1 treatment-related AE; the most common were fatigue (*n* = 7) and pruritus (*n* = 7). Seven patients had G 3/4 treatment-related AEs. No patients discontinued due to treatment-related AEs, and there were no treatment-related deaths. ORR was 13.3% (2 CR, 2 PR), including 3 platinum-resistant patients. Median PFS was 1.8 months (95% CI 1.0–1.9), median OS was 7.9 months (95% CI 3.2–15.8), and 12-month OS rate was 41.7% (95% CI 23.3–59.2). This study indicates that durvalumab in combination with tremelimumab exhibited a tolerable safety profile and promising activity in pretreated ED-SCLC. Responses were durable and seen in both platinum-sensitive and platinum-resistant cases [44]. Additional studies examining durvalumab in SCLC are still ongoing.

## Targeted therapy

### Veliparib

Poly (ADP-ribose) polymerase (PARP) is a family of enzymes involved in DNA damage repair. Overexpression of PARP has been linked to drug resistance and the ability of cancer cells to withstand genotoxic stimuli [45]. Compared to normal lung epithelial cells and other histologic subtypes of lung cancer, the PARP enzyme is highly expressed in SCLC [46]. The small-molecule PARP inhibitor veliparib enhanced the cytotoxic effect of standard chemotherapy agents and radiation in vitro and in vivo preclinical models of SCLC [47, 48]. Temozolomide (TMZ) is an oral alkylating agent that produces O6-alkyl-guanine lesions in DNA. Lesions induced by TMZ are cytotoxic and could trigger apoptosis [49, 50]. Previously, clinical data demonstrated the antitumor activity of TMZ in patients with relapsed SCLC [51]. A phase II study of TMZ in combination with veliparib or placebo was conducted in patients with recurrent SCLC (Table 2). A total of 104 enrolled patients were randomly assigned 1:1 to oral TMZ + veliparib or TMZ + placebo. ORR was significantly higher in patients treated with TMZ + veliparib than in the TMZ + placebo group (39% vs. 14%; *P* = 0.016). However, there was no significant improvement in median PFS between TMZ + veliparib and TMZ + placebo groups (3.8 vs. 2.0 months; *P* = 0.39). OS was also not significantly different (8.2 vs. 7.0 months; *P* = 0.50). Interestingly, PFS and OS were prolonged in patients with schlafen family member 11-positive (SLFN11) tumors when treated with TMZ + veliparib (PFS 5.7 vs. 3.6 months; *P* = 0.009; OS 12.2 vs. 7.5 months; *P* = 0.014). G 3/4 thrombocytopenia and neutropenia more commonly occurred in TMZ/veliparib (50% vs. 9% and 31% vs. 7%, respectively) (Table 4) [52]. A phase II study evaluating the combination of veliparib or placebo with cisplatin and etoposide in untreated, extensive stage SCLC patients also demonstrated modest improved efficacy. A total of 128 patients received treatment randomized 1:1 to receive cisplatin and etoposide together with veliparib or placebo. ORR was 71.9% vs. 65.6% for veliparib and placebo groups, respectively. Median PFS was 6.1 and 5.5 months, respectively, while median OS was 10.3 vs. 8.9 months, respectively. The following G ≥ 3 hematology toxicities were more frequent in the CE+ veliparib arm than the CE + placebo arm: CD4 lymphopenia (8% vs. 0%, respectively; *P* = 0.06) and neutropenia (49% vs. 32%, respectively; *P* = 0.08), but treatment delivery was comparable (Table 4) [53].

**Table 2:** Completed targeted therapy clinical trials in ES-SCLC

Phase	Study	Treatment arms	Patients (*n*)	ORR (%)	PFS (months)	OS (months)
First line						
II	ECOG-ACRIN 2511	Veliparib + etoposide + cisplatin vs. placebo + etoposide + cisplatin	128	71.9 vs. 65.6 (*P* = 0.57)	6.1 vs. 5.5 (HR = 0.75; *P* = 0.06)	10.3 vs. 8.9 (HR = 0.83; *P* = 0.17)
Relapsed						
II	NCT01638546	Veliparib + temozolomide vs.placebo + temozolomide	104	39 vs. 14 (*P* = 0.016)	3.8 vs. 2.0 (*P* = 0.39)	8.2 vs. 7.0 (*P* = 0.50)
II	NCT02454972	Lurbinectedin (PM01183)	68	39.3	4.1	11.8
II	TRINITY	Rovalpituzumab tesirine	177	16	4.1^a^	5.6
II	ALTER 1202	Anlotinib vs. placebo	120	71.6 vs. 13.2^b^	4.1 vs. 0.7 (HR = 0.19; *P* < 0.0001)	7.3 vs. 4.9 (HR = 0.53; *P* = 0.0210, not yet mature)

^a^*DOR* duration of response

^b^*DCR* disease control rate

### Rova-T

Delta-like ligand 3 (DLL3) is normally expressed at low levels in normal tissue but exhibits very high expression in tumors of neuroendocrine origin with more than 80% of SCLC samples showing high expression [54, 55]. An antibody-drug conjugate, rovalpituzumab tesirine (Rova-T), was designed to target DLL3 expressed on SCLC cells and to induce cell death through its cytotoxic payload following internalization into the cytoplasm of the cell. Rova-T was tested in 61 patients with recurrent SCLC. There was a promising signal of efficacy with 25% (15/61) of patients achieving a CR or PR and 72% achieving at least disease stability. DLL3 was highly expressed in more than 50% of patients, and 12 out of these 22 patients achieved a complete or partial response. The clinical benefit rate was 98%, and the median overall survival was 8 months [56]. However, a larger phase II study of Rova-T as a third-line treatment for recurrent SCLC showed far more modest evidence of efficacy with ORR of only 16% (Table 2). The most common treatment-related adverse events were fatigue (38%), photosensitivity reaction (36%), pleural effusion (32%), peripheral edema (31%), decreased appetite (30%), nausea (26%), dyspnea (25%), thrombocytopenia (25%), constipation (22%), vomiting (17%), anemia (17%), hypoalbuminemia (16%), and cough (16%). G 3 and higher severe toxicities ≥ 5% were thrombocytopenia (11%), photosensitivity reaction (7%), and pleural effusion (5%) (Table 4) [57]. Preliminary results of an interim analysis of a phase III trial of Rova-T vs. topotecan in the second line also indicated that Rova-T is not superior to topotecan with a recommendation for trial discontinuation by the independent data safety committee.

### Anlotinib

Anlotinib is an oral tyrosine multikinase inhibitor that targets the vascular endothelial growth factor receptor (VEGFR), platelet-derived growth factor receptor (PDGFR), fibroblast growth factor receptor (FGFR), c-Kit, and other targets. It inhibits both tumor angiogenesis and tumor growth [58] and is an approved treatment for advanced NSCLC by the Chinese Food and Drug Administration (CFDA) based on the ALTER 0303 study [59]. Anlotinib is currently undergoing careful exploration as a treatment option for SCLC, soft tissue sarcoma, colorectal cancer, and other tumor types [60]. The results of a phase II clinical trial (ALTER 1202) of anlotinib as a third line or beyond treatment in SCLC were recently reported (Table 2). The randomized, double-blind, placebo-controlled, multicenter study enrolled a total of 120 SCLC patients. Patients were randomly assigned in a 2:1 ratio to receive anlotinib (*n* = 82, 12 mg once daily orally, 2 weeks on and 1 week off) or placebo (*n* = 38). The primary endpoint was PFS, and secondary endpoints included OS, ORR, disease control rate (DCR), quality of life, and safety. Median PFS was 4.3 months in the anlotinib group vs. 0.7 months in the placebo group (HR = 0.19, *P* < 0.0001). Median OS was 7.3 monthS and 4.9 months for anlotinib and placebo groups, respectively. DCR was also superior for the anlotinib arm at 71.6% vs. 13.2% in the placebo group. The observed toxicity profile in this study was similar to a previous study of anlotinib in NSCLC. G 3–4 toxicity was slightly higher than in the placebo group with on target toxicity of bleeding in the form of hemoptysis being the most serious complication observed on the study, which occurred in four patients with only one case requiring treatment intervention [61].

## Ongoing studies

There are many ongoing clinical trials for ES-SCLC [62], some of which are shown in Table 3. For first-line treatment, REACTION is a phase II study evaluating outcomes of pembrolizumab with or without standard chemotherapy. CASPIAN is another phase III study performed on first-line treatment for SCLC. The treatment arms included durvalumab + tremelimumab + cisplatin/carboplatin + etoposide vs. durvalumab + cisplatin/carboplatin + etoposide vs. cisplatin/carboplatin + etoposide. These two studies are both still actively recruiting. For maintenance treatment after first-line treatment, the CheckMate-451 study was performed with nivolumab vs. nivolumab + ipilimumab vs. placebo arms. This study has currently stopped recruiting, and Bristol-Myers Squibb announced that CheckMate-451 did not meet its primary endpoint of OS.

**Table 3:** Ongoing studies of immunotherapy in extensive stage small cell lung cancer

Phase	Study	Treatment arms	ClinicalTrials.gov identifier	Estimated primary completion date
First line				
II	REACTION	Cisplatin/carboplatin + etoposide + pembrolizumab vs. cisplatin/carboplatin + etoposide	NCT02580994	August 2020
III	CASPIAN	Durvalumab+ tremelimumab+ cisplatin/carboplatin + etoposide vs. durvalumab+ cisplatin/carboplatin + etoposide vs. cisplatin/carboplatin + etoposide	NCT03043872	September 2019
Maintenance				
III	CheckMate-451	Nivolumab vs. nivolumab + ipilimumab vs. placebo	NCT02538666	October 2018
Relapsed				
I/II	CheckMate-331	Nivolumab vs. topotecan vs. amrubicin	NCT02481830	August 2018
II	Winship3112-15	Tremelimumab + durvalumab vs. tremelimumab + durvalumab + radiation	NCT02701400	January 2020
II	AFT-17	Pembrolizumab vs. topotecan	NCT02963090	May 2019
I/II	CA001-030	BMS-986012 vs. BMS-986012 ± nivolumab	NCT02247349	October 2019
I/II	MEDIOLA	Durvalumab + olaparib vs. durvalumab + olaparib + bevacizumab	NCT02734004	March 2023

For relapsed treatment, the CheckMate-331 study contained nivolumab vs. topotecan vs. amrubicin arms. Bristol-Myers Squibb also announced that this phase III study failed to meet its primary endpoint of OS. Other studies focused on relapsed treatment, for instance, the Winship3112-15 study is comparing tremelimumab and durvalumab with and without radiation therapy, the AFT-17 study is examining pembrolizumab and topotecan, and CA001-030 is a phase I/II study to explore the safety and outcome of BMS-986012 in relapsed SCLC. In addition, MEDIOLA is a phase I/II study of durvalumab in combination with olaparib in patients with advanced solid tumors, including SCLC. AEs of the ongoing studies are still unknown, however, AEs of the majority of completed studies are shown in (Table 4).

**Table 4:** Main grade 3 or higher treatment-related AEs in the present article

Study	Main grade 3 or higher toxicities (over 10%)
NCT01331525	Neurological AEs (10.3%), diarrhea (48.7%), neutrophil count decrease (23.1%), anemia (15.4%), infection (28.2%), and sepsis (10.3%).
CA184-041	ALT (18%) and AST (13%) in concurrent arm vs. fatigue (12%), arthralgia (10%), diarrhea (10%), neutropenia (10%), and anemia (10%) in phased arm.
CA184-156	Neutropenia (24%) and anemia (11%) in chemotherapy plus ipilimumab arm vs. neutropenia (14%) in chemotherapy plus placebo arm.
Impower-133	Neutropenia (22.7%), anemia (14.1%), decreased neutrophil count (14.1%), and thrombocytopenia (10.1%) in chemotherapy plus atezolizumab arm vs. neutropenia (24.5%), anemia (12.2%), and decreased neutrophil count (16.8%) in chemotherapy plus placebo arm.
NCT02359019	Most common adverse events were fatigue, nausea, cough, and dyspnea. One patient developed atrioventricular conduction block and one patient type 1 diabetes. No grade 3 to 5 treatment-related AEs was over 10% of the participants.
CheckMate-032	Two patients who received nivolumab 1 mg/kg plus ipilimumab 3 mg/kg and one patient who received nivolumab 3 mg/kg plus ipilimumab 1 mg/kg died from treatment-related adverse events. No grade 3 to 5 treatment-related AEs was over 10% of the participants.
KEYNOTE-028	Treatment-related AEs were seen in 16 (66.7%) of 24 patients. Two patients experienced grade 3 to 5 treatment-related AEs. No grade 3 to 5 treatment-related AEs was over 10% of the participants.
KEYNOTE-158	Treatment-related AEs occurred in 63 patients (59%) and led to 4 discontinuations and 1 death (pneumonia). No grade 3 to 5 treatment-related AEs was over 10% of the participants.
NCT02261220	Twenty patients (67%) reported ≥ 1 treatment-related AE (TRAE); the most common were fatigue (*n* = 7 [23%]) and pruritus (*n* = 7 [23%]). Seven patients (23%) had grade 3/4 TRAEs. No patients discontinued due to TRAEs, and there were no treatment-related deaths.
ECOG-ACRIN 2511	Neutropenia (49%), anemia (19%), leukopenia (19%), and hyponatremia (12%) in chemotherapy plus veliparib arm vs. neutropenia (32%), anemia (12%), and leukopenia (14%) in chemotherapy plus placebo arm.
NCT01638546	Leukopenia (24%), lymphopenia (20%), neutropenia (31%), and thrombocytopenia (50%) in veliparib plus temozolomide arm vs. lymphopenia (26%) in temozolomide plus placebo arm.
NCT02454972	Neutropenia grade (44%)
TRINITY	Thrombocytopenia (11%)
ALTER 1202	Grade ≥ 3 TRAEs occurred in 29 (35.8%) of patients in anlotinib arm and 6 (15.4%) in placebo arm.

## Conclusions/expectations

Immunotherapy is the most promising SCLC treatment in recent years [63, 64]. Based on the CheckMate-032 study, nivolumab was approved by the Food and Drug Administration (FDA) for recurrent SCLC, making it the first FDA-approved third-line treatment for SCLC. Atezolizumab in combination with chemotherapy as a first-line treatment also demonstrated improved efficacy in the IMpower133 study. This is the first phase III study to achieve an improvement OS in more than 30 years for extensive stage SCLC. Despite immunotherapy having become a primary component of SCLC treatment, there are still many challenges, such as efficacy being modest and limited to a small subset of patients [65]. Identifying predictive biomarkers for selecting the patient subgroup most likely to benefit from this treatment strategy is an area of significant unmet need [66].

Immunotherapy combined with radiotherapy represents a new method for treating SCLC. The PACIFIC trial in NSCLC demonstrated that PFS and OS were significantly longer with durvalumab than with placebo, especially in patients with PD-L1 TC ≥ 1%, while safety was similar between the groups [67]. Since SCLC is sensitive to radiotherapy and concurrent chemotherapy, radiotherapy is the standard first-line treatment for limited stage SCLC. Immunotherapy applied concurrently with radiotherapy or immunotherapy applied after concurrent chemotherapy and radiotherapy might further improve ORR and prolong survival time.

Although targeted therapy has dramatically changed our approach to treating NSCLC, similar breakthroughs have not materialized for SCLC. The efficacy of anlotinib in heavily pretreated recurrent SCLC is a potential light at the end of the tunnel, but these initial results require further validation before this agent can become a standard treatment option for SCLC patients. Furthermore, initial promise with an antibody drug conjugate targeting DLL3 now appears somewhat illusory in the face of larger prospective studies that failed to replicate the efficacy of Rova-T in relapsed SCLC. Strategic pairing of DNA repair inhibitors, such as PARP inhibitors, with standard chemotherapy agents could lead to improvements in efficacy based on the results of early phase II study findings.

In addition to the emerging drugs and clinical studies mentioned above, there are still many more new drugs and treatment combinations that have conducted preclinical studies or are in early stages of clinical development. New immune drugs can be broadly classified as checkpoint inhibitors (other than PD-L1/PD1 inhibitors), CTLA-4 antibodies, agonists of costimulatory receptors, T cells manipulators, oncolytic viruses, and therapies directed at other cell types and vaccines [65]. Additionally, many treatment combinations are being explored with new drugs, some of which have provided strong rationale for further clinical trials in SCLC, such as olaparib and the WEE1 inhibitor AZD1775 [68]. However, these preclinical studies provide limited information and lack of favorable clinical evidence; therefore, we did not further elaborate on them.

**Fig. 1:**
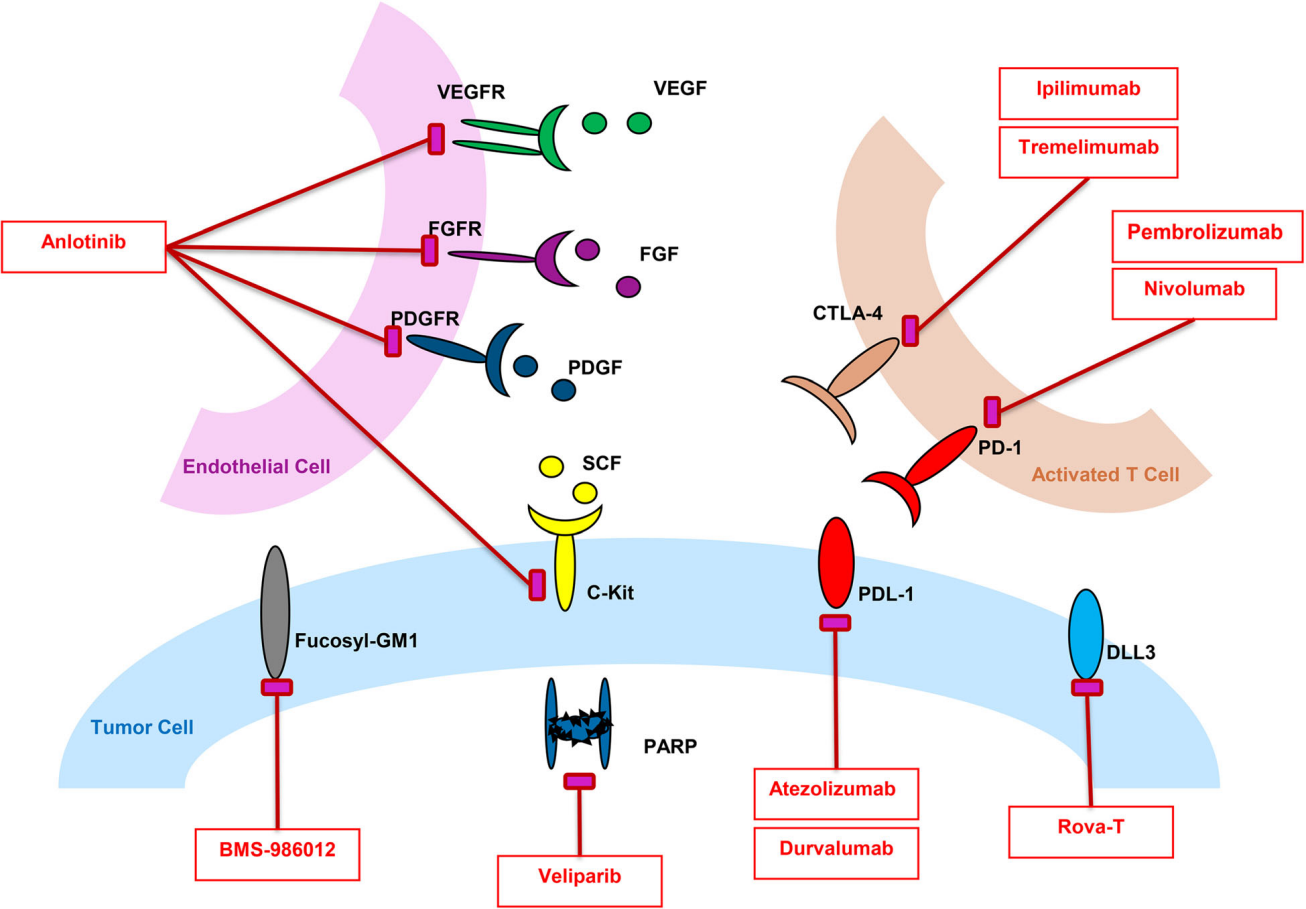
Mechanisms of action for targeted agents. VEGFR, vascular endothelial growth factor receptor; PDGFR, platelet-derived growth factor receptor; FGFR, fibroblast growth factor receptor; DLL3, delta-like protein 3; PARP, poly (ADP-ribose) polymerase; PDL-1, programmed death ligand 1; PD1, programmed death 1; CTLA-4, cytotoxic T lymphocyte antigen-4

**Fig. 2:**
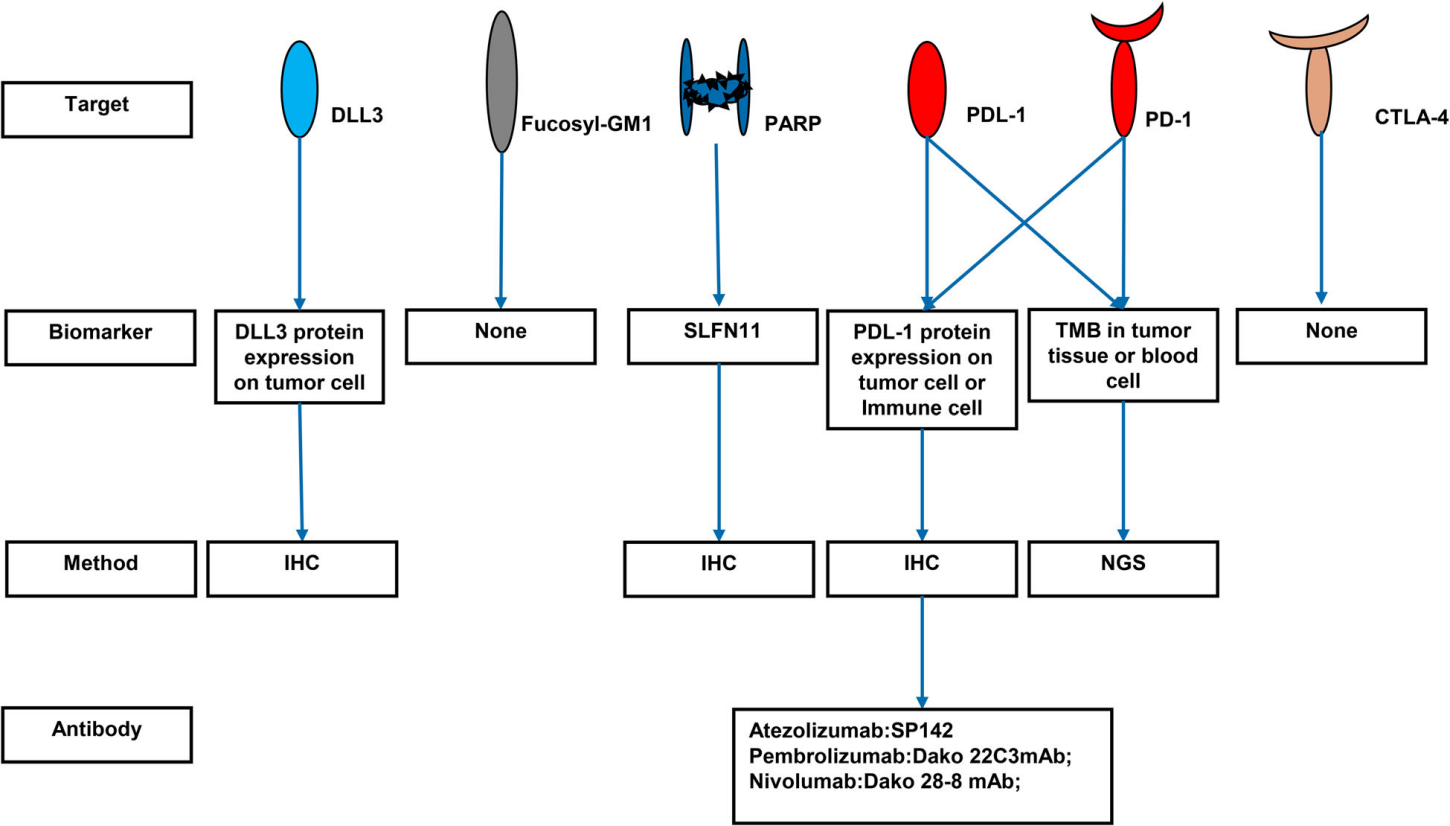
Targets and biomarkers for targeted therapy. DLL3, delta-like protein 3; PARP, poly (ADP-ribose) polymerase; PDL-1, programmed death ligand 1; PD1, programmed death 1; CTLA-4, cytotoxic T lymphocyte antigen-4; SLFN11, schlafen family member 11; TMB, tumor mutation burden; IHC, immunohistochemistry; NGS, next-generation sequencing

Given that immunotherapy drugs, targeted therapy drugs, and chemotherapy drugs act on different targets and cells (Fig. 1), synergistic or combined treatment of these drugs may achieve greater therapeutic effects at the cost of similar side effects. However, the success of this strategy will require the use of validated biomarkers to select patients most likely to benefit from such a strategy (Fig. 2). Overall, there seems to be hope on the horizon for patients with SCLC after many decades of negative trials and promising but failed strategies that did not improve patient outcomes.

## Abbreviations

AE: Adverse event
ASCO: American Society of Clinical Oncology
BORR: Best overall response rate
CI: Confidence interval
CR: Complete remission
CTLA-4: Cytotoxic T lymphocyte antigen-4
DCR: Disease control rate
DLL3: Delta⁃like protein 3
FDA: Food and Drug Administration
HR: Hazard ratio
NCCN: National Comprehensive Cancer Network
NSCLC: Non-small cell lung cancer
ORR: Objective response rate
OS: Overall survival
PARP: Poly (ADP-ribose) polymerase
PCI: Prophylactic cerebral irradiation
PD-1: Programmed death 1
PD-L1: Programmed death ligand 1
PFS: Progression-free survival
PR: Partial remission
Rova-T: Rovalpituzumab tesirine
SCLC: Small cell lung cancer
TKI: Tyrosine kinase inhibitor
TMB: Tumor mutation burden
TMZ: Temozolomide
